# Oral Health Impact Profile in Celiac Patients: Analysis of Recent Findings in a Literature Review

**DOI:** 10.1155/2018/7848735

**Published:** 2018-10-24

**Authors:** Gabriele Cervino, Luca Fiorillo, Luigi Laino, Alan Scott Herford, Floriana Lauritano, Giuseppe Lo Giudice, Fausto Famà, Rossella Santoro, Giuseppe Troiano, Gaetano Iannello, Marco Cicciù

**Affiliations:** ^1^Department of Biomedical and Dental Sciences and Morphological and Functional Imaging, Messina University, 98100 Messina, Italy; ^2^Multidisciplinary Department of Medical-Surgical and Odontostomatological Specialties, University of Campania “Luigi Vanvitelli”, 80121 Naples, Italy; ^3^Department of Maxillofacial Surgery, Loma Linda University, Loma Linda, CA 92354, USA; ^4^Department of Human Pathology, University of Messina, 98100 Messina, Italy; ^5^Departments of Clinical and Experimental Medicine, University of Foggia, 71121 Foggia, Italy

## Abstract

The increment of recording atypical oral manifestation in young patients often related to systematic disease is today a challenge for the therapists. Sometime, the presence of tooth enamel lesions correlated with soft tissue lesions is just a symptom or a trigger sign for a deeper and undetermined disease. Recently, high impact has been developed toward the influence of the diet as a controlled and modifiable factor in patients affected by celiac pathologies. The celiac disease (CD) is a chronic immune-mediated disorder triggered by the ingestion of gluten that appears in genetically predisposed patients. Gluten is a proline-rich and glutamine-rich protein present in wheat (gliadin), barley (hordein), and rye (secalin). The gluten-free diet (GFD) seems to better influence the oral health status of the CD patients. For this reason, the main objective of this revision was to analyze the international data highlighting the relationship between celiac patients and the oral health impact profile. A comprehensive review of the current literature was conducted according to the PRISMA guidelines by accessing the NCBI PubMed database. Authors conducted the search of articles in the English language published from 2008 to 2018. The first analysis with filters recorded 67 manuscripts accordingly with the selected keywords. Finally, a number of 16 appropriate published papers were comprehended in the review. The studies were different in terms of the structure, findings, outcomes, and diet quality evaluation, and for this reason, it was not possible to accomplish a meta-analysis of the recorded data. This manuscript offers some observational evidence to justify the advantages of gluten-free diets related to a better oral health status in the patients involved.

## 1. Introduction

Oral health is today considered one of the fundamental parameters related to the patient's general health and behavior. Oral health status allows individuals to run their daily activities (mastication, articulation, and socialization) without any pain, discomfort, and restriction. The patients' quality of life (QoL) is a caption currently applied in the medicine field to refer to social well-being and the effects of therapy on cancer patients. Specifically, in the dental practice, QoL, as it connected to the oral health, has only been recently employed [1–7].

The patients' general health condition is related to having no problems or diseases on all the anatomical structures, even involving the oral cavity functions or aesthetics. Today, great attention is focused on the prevention and maintenance of high standard of oral hygiene and control; however, some pathologies may be connected with systemic disease and not affect the oral cavity structures directly [3, 7–10].

Nowadays, even the current standard of performing diagnosis in oral lesions like teeth enamel defects or soft tissues and tongue lesions may be related to local affection or trauma; a deep knowledge of the patient anamnesis and clinical history is required in order to evaluate possible hidden causes strictly related to the diet or general health status. Therefore, it is well documented how many systemic diseases are somehow related to many oral manifestations and influence the individual quality of life [4, 9, 11–13].

Celiac disease (CD) is a long-term autoimmune disorder that affects the small intestine; this is caused by a constant intolerance to gluten proteins in genetically susceptible individuals. CD is caused by a reaction to gliadins and glutenins found in wheat. These protein-based factors may be responsible for a toxic event on the intestinal mucosa in genetically receptive subjects by triggering an immune-mediated reaction, related to the common villous atrophy and lymphocyte infiltrate in the small intestine mucosa recorded in CD. Common oral and dental manifestations of CD include mouth ulcers, in particular, recurrent aphthous ulcers, and dental enamel defects [13–16].

However, even if great important steps have been done in the field of quick diagnosis, CD is still not promptly diagnosed, because recently, the typical form of CD, characterized by modified absorption and gastrointestinal signs, is less recurrent compared with the atypical forms, often asymptomatic and involving extraintestinal clinical manifestations. A multidisciplinary evaluation and approach between clinicians, pediatricians, and gastroenterologists should be performed in order to underline all the extraintestinal possible manifestations of CD and to make an early diagnosis; recurrent aphthous ulcers, previously mentioned, could provide another clue to the possible presence of this disorder [17–20].

Numerous published papers underlined how specific oral signs and symptoms can be classified as risk factor signals for CD; however, only the internal specialist can perform the diagnosis, evaluate the presence of specific celiac antibodies, and demonstrate intestinal mucosa damages. However, the topic is still debated, and currently, the right frequency of these oral manifestations in potential celiac patients has not yet been classified and recorded [21–23].

However, it is widely recognized that, among these atypical signs of CD, there are certain oral manifestations which are surely interwoven to CD: tooth enamel lesions and defects, frequent aphthous stomatitis, delayed tooth eruption, multiple caries, angular cheilitis, atrophic glossitis, dry mouth, and burning tongue. For this reason, dentists and the first dental visits play a fundamental role in detecting symptoms related to CD and for the next medical treatments [23, 24].

About the treatments and the prevention of such oral manifestations, recently published investigations underlined how the gluten-free diet may favor an improvement of the general health condition of celiac and no celiac patients. Moreover, numerous epidemiological and clinical studies recorded a decrease of oral CD manifestations connected with the gluten-free diet of the involved patients [25–33].

The aim of the present revision is to examine the data of the last ten years' literature about oral manifestations of celiac disease and how gluten-free diet may definitely influence the conditions of the oral health patients.

## 2. Methods of Screening

### 2.1. Protocol Development and Online Information Recording

The inclusion parameters for the current research were collected in a protocol and then submitted in advance and documented in CRD York website PROSPERO, a global prospective catalogue of revision manuscripts. The criteria and the formal structure of the present revision can be searched online with the CRD id and code: application number: CRD 92075.

The documents collected in the present paper followed the Preferred Reporting Items for Systematic Review and Meta-analysis (PRISMA) statement accordingly [34, 35].

### 2.2. Outcome Query

The following question was sentenced and structured according to the (PICO) study design:

- How can the celiac disease influence the oral health status of the patients?

### 2.3. Searches

**Figure 1:**
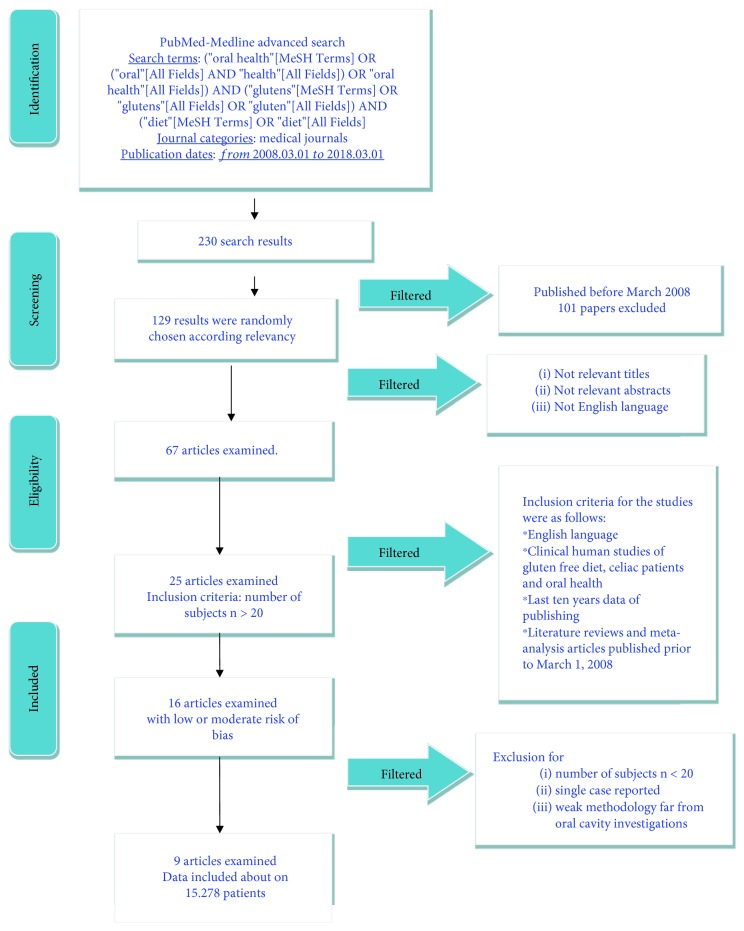
PRISMA flow chart diagram, including all the screening methodology, and revision progresses.

The PubMed-Medline resource database was applied through advanced researches. The keywords and search inquiries used during the primary stage were as follows: “oral health gluten-free diet.” Additional manually selected articles were included regarding the eligibility criteria.  Figure 1 represents the flow diagram of the selected studies according to PRISMA guidelines and following the criteria for the investigated paper choice. Web searching and researches by hand were then executed in the field of gastroenterology, dentistry, and medicine journals, finding different international journals. The search was restricted to English language manuscripts.

### 2.4. Data Recorded from the Selected Manuscripts

The Medical Subject Headings (MeSH) was used for finding the keywords used in the present revision. The selected keywords (“oral health”[MeSH Terms] OR (“oral”[All Fields] AND “health”[All Fields]) OR “oral health”[All Fields]) AND (“glutens”[MeSH Terms] OR “glutens”[All Fields] OR “gluten”[All Fields]) AND (“celiac”[MeSH Terms] OR “diet”[All Fields]) were written in the selected database.

### 2.5. Selections of the Papers

Three separate researchers, of three Italian universities (Messina, Foggia, and Naples Universities), singularly screened the full-text manuscripts for deciding inclusion and exclusion criteria. Reviewers compared decisions about criteria, parameters, and selected papers. During the step of reviewing the manuscripts, a complete independent two-fold revision was undertaken.

Reviewers compared their results and data. A fourth qualified chief reviewer (Loma Linda University) was then contacted when agreement could not be obtained at the first step of paper revisions.

The papers recorded in the present revision highlighted clinical researches over celiac patients printed in the English style. Letters, editorials, case reports, animal studies and degrees, and PhD thesis were not included in the revision process.

### 2.6. Studies Involved in the Revision

The design of recording data involved all human prospective and retrospective clinical studies, split-mouth cohort studies, case–control papers, and case series manuscripts, published between March 2008 and March 2018, about gluten-free diet, oral disease, and celiac patients.

### 2.7. Exclusion and Inclusion Criteria

The applied inclusion criteria for the studies were as follows:

- English language
- Clinical human studies of gluten-free diet, celiac patients, and oral health
- Last ten year data of publishing
- Literature reviews and meta-analysis articles published prior to March 1, 2008

The following types of articles were excluded as follows:

- In vivo/in vitro studies
- Studies of testing medication and/or new treatment methodologies
- Studies of cancer in locations other than mentioned
- Studies not relevant to our selected diagnostic methods
- Animal studies
- No availability to the title or summary not in English words

### 2.8. Data Recording Design

Following the initial literature screening, all the paper titles were evaluated in order to delete irrelevant publications, case reports, and animal studies. Then studies were excluded based on data obtained from reading the summaries. The last step of screening involved reading the full texts to determine each study's selection following the inclusion and exclusion criteria. (Figure 1).

### 2.9. Risk of Bias Assessment

The quality of all involved texts was assessed during the data extraction process. The quality appraisal involved evaluating the methodological elements that might influence the outcomes of each study.

Each reviewer evaluated the level of possible bias risk during the information taking out method. This revision work was made accordingly with the Cochrane Collaboration's double tool for determining risk of bias and PRISMA rules [34, 35].

Differences in risks of bias can help explain variation in the results of the studies included in a systematic review (i.e., explain heterogeneity of results). More rigorous studies are more likely to yield results that are closer to the truth.

Risk of bias (e.g., the absence of information or selective reports on variables of interest) was assessed on the study level. The risks were indicated as lack of precise information of interest related to the keywords selected. Finally, the researches selected for the revision were then recorded in modest, moderate, significant, and unclear risk.

## 3. Outcomes

### 3.1. Paper Recording and Possible Bias

The PRISMA flow diagram describes the revision steps for screening the papers and reaching the selected ones (Figure 1). The initial web and hand searches performed on PubMed-Medline and Oral Sciences Source produced a number of 230 findings. 101 references were not involved in the revision because they were printed before March 1, 2008. Then another 62 papers were not selected for the data because they were not available on full text. 67 papers were discovered on full-text form, 25 of which were merged in this work, and then after final screening, a total of 9 full-text papers.

During the last deep screening section, from the last 16 manuscripts, some researches were excluded because they were recorded as a unique case report (*n* = 2) or not significant design study or procedures were far from the topic (*n* = 5). So finally, 9 papers were recorded and screened in this revision paper.

No meta-analyses could be performed due to the heterogeneity between the studies (different study designs, control groups, and observation periods) Table 1. The possible risk of bias was considered for each selected papers. The final number of the selected papers was limited from 25 full-text papers to 9. The inclusion criteria were really restrictive, and for this reason also, the risk of bias was low. Ten types of research were evaluated as having minor risk of bias [36–44] whereas another seven were classified as moderate risk [45–51].

The present investigation of the data extracted from researches printed in English only could detect a publication bias. About possible bias, some of the selected papers did not specify the inclusion criteria of the patient selection. Another key parameter that can be assumed as bias is related to the evaluation of the clinical condition for selecting the patient. Moreover, data recorded from the eight studies pointed out the heterogeneity of the research methods, selections of the patients, and therapeutic options.

## 4. Results

The present systematic review discovered gluten-free diet is associated with oral health status of celiac patients. Due to high heterogeneity of the researches, it was not realizable to do meta-analysis for comparing the data of the selected papers. Due to poor material, it is not possible to establish specific oral health status related to diet or systemic diseases like celiac patients.

Moreover, even wide screening and research have been performed; the inclusion criteria related to the “oral cavity” was really inclusive, and for this reason, it was not possible to state some guidelines that may significantly increase the oral health status of the CD patients just by applying a gluten-free diet. Some papers with low risk of bias [36–44] clearly analyzed the correlation between gluten-free diet and oral health status of celiac patients. However, the data of those researches are not significant and finally suggested some recommendations and not guidelines. Specifically, because the disease involves the gastrointestinal area, the high part of the researches firstly investigated the microbiota related to the anatomical area far from the oral cavity area. Therefore, all the data extracted from the present revision clearly underlined how a diet associated with no gluten may favor high standard of oral health quality delaying gingival oral disease due to the alteration of the oral microbiota.

### 4.1. Oral Soft Tissue Manifestations

Oral soft tissue manifestations in CD-affected patients are reported in literature. Oral manifestations that interest gums such as aphthous ulcers or recurrent aphthosis are correlated to celiac disease-affected patients. These manifestations are more frequent in CD patients than in normal population [38, 39].

### 4.2. Dental Manifestations

Dental hard tissue manifestations in CD patients are various; we can find alterations on the enamel and teeth structure. Some studies report enamel hypoplasia, enamel defect or enamel and dental structure alterations [38–40, 42–44], this condition puts CD patients in condition of discomfort, lowering their general conditions of oral health and expelling them to other debilitating diseases.

### 4.3. Oral Health

Another series of oral manifestations is present in CD patients which does not affect soft tissue or the dental structure. Some studies evaluated the oral health of patients through self-administered tests like the OHIP-14 test (Oral Health Impact Profile 14) or XI test (Xerostomia inventory). Studies reported abnormalities in general oral health like DMFT index (Decayed, Missing, Filled Teeth); some other studies reported anomalies like delayed eruption and parodontal disease [36–42].

## 5. Discussion

The purpose of this review was to systematically overview published studies restricted to oral health and gluten-free diet in order to evaluate how a diet without gluten may influence the oral health status of celiac patients, following the brief report of the 16 papers classified with moderate and low risks of bias.

Spinell et al. [36] recently investigated whether celiac disease was associated with periodontitis or periodontal diseases among a population of US adult patients. In this large research, the National Health and Nutrition Examination Survey (NHANES) authors between 2009 and 2012 enrolled about 6661 subjects with full-mouth periodontal examination and serological testing for antitissue transglutaminase (tTg) and antiendomysial (EMA) antibodies. CD was defined as (i) self-reported physician diagnosis while on a gluten-free diet or (ii) tTg levels > 10.0 U/ml and positive EMA results. Positive serology without self-reported diagnosis was defined as undiagnosed CD (UdxCD). Authors concluded how CD is associated with modestly lower levels of mean periodontal disease but was not associated with periodontitis in a significant way. Larger studies are necessary to enhance precision and strengthen conclusions.

The oral health status and the xerostomia of celiac patients were investigated by van Gils et al. [37] in a study involving a population of about 740 patients with CD and 270 comparison participants. The Oral Health Impact Profile 14 (OHIP-14) and Xerostomia Inventory (XI) were screened and recorded. This study showed that oral health problems are more commonly experienced in adult patients with CD than in the comparison group. Collaboration between dentists and gastroenterologists is recommended to increase detection of undiagnosed CD.

De Angelis et al. [45] analyzed how the oral and intestinal bacteria metabolize dietary components, affecting human health by producing harmful or beneficial metabolites, which are involved in the incidence and progression of several intestinal-related and nonrelated diseases. Moreover, the authors stated how dietary regimens with fibers are the most effective to benefit the metabolism profile, and a profitable use of diet is fundamental in order to provide benefits to human health, both directly and indirectly, through the activity of the gut microbiota.

In a different revision paper, Cenit et al. [46] evaluated how in mature normal subjects, the GFD is connected with a low intake of complex polysaccharides caused shifts in the gut microbiota structure. Therefore, the authors concluded that microbiota imbalances have been recorded not only in untreated CD subjects but also in patients following a GFD. Moreover, typical bacterial strains isolated from subjects with active and nonactive CD have been shown to increase virulence features. These alterations may be significant for increasing CD pathogenesis by contributing to the disease onset.

Galipeau and Verdu [47] recorded significant findings in their review underlining and effective evidence between intestinal dysbiosis and CD; however, the main limit of the present investigation was related to the analysis of the manuscripts classified in the revision. It was determined evidence demonstrating causality is lacking. Therefore, it remains unclear whether general changes in microbial composition or the presence or absence of particular members of the microbiota play a direct role in CD pathogenesis, and so the diet is not fundamental in the CD developments.

Rivera et al. [38] in their research studied how CD continues to be an unsolved puzzle and a much-debated topic in the recent literature. Knowing the important health implications that CD can have, not only in an individual's health but also in the overall quality of life of these individuals and their families, is of vital importance. As clinicians, it is very important to be aware of the potential presentations, especially in terms of oral health, that CD can have and the consequences it can lead to in overall health status. When suspected, it is extremely important to refer the patient to a gastroenterologist for further evaluation, diagnosis, and, in the case of a positive work up, initiation of treatment with a GFD.

Shteyer et al. [39] made an interesting report studying the oral health status and quality in relation to GFD in children with CD. The results showed that newly diagnosed children with CD have more dental plaque and caries than the control groups, and children receiving GFD had lower dental plaque and better oral hygiene. These results should raise pediatric gastroenterologists' awareness toward oral health–related issues in children with CD. However, the data of the present investigation were not clear if the enamel defects were genetic or due to the low oral health conditions of the CD patients.

Mina et al. [40] in their study highlighted the main difference among CD children who did or did not comply with a gluten-free diet and control children are the presence of PMNs in the oral mucosa and protein salivary patterns; these findings could be considered as markers for CD, in conjunction with other signs and symptoms. The GFD seems to improve the oral health quality reducing the gingival inflammation.

Tsami et al. [41] inspected the factors that influence the oral hygiene and the periodontal treatment needs of children and adolescents with celiac disease (CD). It was found that the periodontal treatment need of children and adolescents with CD correlated with factors that related to the presence of a second medical condition and to the personal oral hygiene habits. CD seems to not have significant influence on the oral health status of the CD adolescents. Additionally, the oral hygiene level and periodontal status of children with CD do not have any specific characteristics, but they have similarities to the oral hygiene level and periodontal status of the children of the general population.

da Silva et al. [48] made a brief review of the literature about CD and analyzed a clinical case, and for this reason, this paper was not included in the final 9 papers. However, the management of the case was typical. A 39-year-old woman reported the presence of many symptoms. She also noted the appearance of symptomatic lesions in the mouth. These lesions had a mean duration of a month and occurred in any region of the oral mucosa, particularly on the tongue. Topical treatment was instituted for the oral lesions with immediate relief of the symptoms. The diagnosis of celiac disease was established by means of a medical clinical exam. A multidisciplinary approach and management with the involvement of a gastroenterologist and other health professionals, such as dentists, are important for diagnosing the disease and guiding the patient with celiac disease to achieve a good quality of life.

Francavilla et al. [49], thanks to advances in understanding the immunopathogenesis of CD, have proposed different kinds of treatment options alternative to the GFD. Some of these therapies try to decrease the immunogenicity of gluten-containing grains by modifying the grain itself or by applying oral enzymes to break down immunogenic peptides that usually remain intact during digestion.

Bascuñán et al. [50] evaluated how the only effective and safe treatment of celiac disease continues being the so-called gluten-free diet (GFD). Although GFD poses difficulties to patients in family, social, and working contexts, deteriorating his/her quality of life. The diet must be not only free of gluten but also healthy to avoid nutrient, vitamins, and mineral deficiencies or excess. Overweight/obesity frequency has increased. Authors concluded how nutritional education by a trained nutritionist is of great relevance to achieving long-term satisfactory health status and good compliance.

Theethira and Dennis [51] underlined how it is important to have regular follow-up visits and lab work to detect and treat nutritional deficiencies after initiation of the GFD. Indisputably, the GFD is the cornerstone of treatment for CD. Keeping these nutritional concerns in mind, a patient with CD can enjoy a healthy, well-rounded diet that improves and maximizes overall health and well-being.

Souto-Souza et al. [42] investigated the relationship between developmental defects of enamel and celiac disease. In their meta-analysis, it was observed how subjects with CD had a significantly higher prevalence of enamel defects matched with healthy people. The most important findings of this paper are that only developmental defects of enamel diagnosed using Aine's method were strictly related to CD. In a sensitivity analysis involving the deciduous, mixed, and permanent dentitions, only individuals with deciduous dentition were observed to have association with the disease. Then patients with enamel developmental defects should be screened for the possibility of having celiac disease.

Sóñora et al. [43], based on previously reported cross-reactivity of antibodies to gliadin with the enamel proteins, amelogenin and ameloblastin, investigated the ability of antigliadin IgG to recognize enamel organ structures. Strong staining of the enamel matrix and of the layer of ameloblasts was observed with serum samples from women with celiac disease. The results strongly advise a pathological position for antibodies to gliadin in enamel defect dentition for both deciduous and permanent teeth, considering that IgG can be transported through the placenta during fetal tooth development. Muñoz et al. [44] in their research classified the pathogenesis of enamel anomalies in permanent teeth of subjects affected by CD. The studies using ELISA and western blotting, for reactivity of sera from patients with CD against gliadin and peptides obtained from enamel, confirm that the antibodies against gliadin generated in patients with CD can react in vitro with an important enamel protein. The involvement of antigliadin serum in the pathogenesis of enamel defects in children with untreated CD can be hypothesized on the basis of these new results (Table 2).

Hypoplasia of the enamel, xerostomia, and oral gingival lesions are the most common symptoms reflected in the recorded manuscripts. The tooth enamel defects can be a clinical sign that can be useful for performing quick diagnosis of CD, but the defect can be managed only by dental conservative treatment, and a GFD cannot modify these clinical conditions. After all, xerostomia and oral ulcers and gingival lesions, other clinical signs of CD disease, can be topically treated by dental care, but in those case, a GFD diet seems to favor an increase of the oral health status of the CD patients.

## 6. Conclusions

Reading the selected papers, it is possible to screen about 15,000 CD patients. Even if this number is large, at the same time, it is not significative and representative, because the studies presented high heterogeneity criteria and methods for evaluations.

## Figures and Tables

**Table 1:** CD and OH parameters recorded in the studies with low risk of bias (CD = celiac disease, PD = parodontal disease, OH = oral health, DDE = developmental defects of enamel, DED = dental enamel defects, and AGA = antigliadin antibodies).

References	Year	Author	Subjects (*n*)	Type of correlation	Result	Statistic
[36]	2018	Spinell et al.	6661	PD and CD	There is no significant correlation between PD and CD.	*P* = 0.67
[37]	2017	van Gils et al.	5522	CD and xerostomia; CD and OH	OHIP-14 and XI tests were performed.	*P* < 0.001 for both
[38]	2013	Rivera et al.	20	CD and enamel hypoplasia; CD and aphthous ulcers; CD and delayed eruption	Oral manifestations. In the long list of clinical signs and symptoms that have been found significantly associated with CD are oral manifestations such as dental enamel hypoplasia, aphthous ulcers, and delayed eruption of teeth.	Reported as significant values, insufficient extrapolated statistical data
[39]	2013	Shteyer et al.	90	CD and enamel defects; CD and aphthous stomatitis, DMFT	A high prevalence of enamel hypoplasia (66%) was found in children with CD. Plaque index was significantly lower in the celiac-treated group.	*P* < 0.05
[40]	2012	Mina et al.	25	CD and OH, enamel alteration	DMFT, enamel alterations, gingival index, and oral hygiene were evaluated in this study.	*P* < 0.0001
[41]	2010	Tsami et al.	35	CD and OH	The periodontal treatment need of children and adolescents with CD was high, most of them needed treatment for gingivitis (60.01%), and only a few subjects had a healthy periodontium (34.29%).	*P* < 0.05
[42]	2018	Souto-Souza et al.	2840	CD and OH; CD and enamel alterations	This meta-analysis indicated a high prevalence of DDE among celiac patients with a significant association of DDE with this population when compared to healthy people.	*P* = 0.014
[43]	2016	Sóñora et al.	21	CD and enamel defects	These results strongly suggest a pathological role for antibodies to gliadin in enamel defect dentition for both deciduous and permanent teeth.	Insufficient extrapolated statistical data
[44]	2012	Muñoz et al.	64	CD and enamel defects	This work describes structural similarities between gliadins and proline-rich enamel proteins and shows that potential cross-reactions of AGA could take place during amelogenesis in untreated patients. Further work is underway to study the biological effects of AGA on enamel formation to evaluate their putative role in the pathogenesis of DED in untreated CD.	*P* = 0.045

**Table 2:** Selected papers in which there is a direct correlation between CD and oral health alterations or disease.

References	Author and year	Subjects (*n*)	Oral health status and symptoms
[36]	Spinell et al. 2018	6661	Periodontitis
[37]	van Gils et al. 2017	5522	Periodontitis and xerostomia
[38]	Rivera et al. 2013	/	Enamel hypoplasia; aphthous ulcers, delayed dental eruption
[39]	Shteyer et al. 2013	90	Enamel defects; aphthous stomatitis
[42]	Souto-Souza et al. 2018	2840	Enamel alterations
[43]	Sóñora et al. 2016	21	Enamel defects
[44]	Muñoz et al. 2012	64	Enamel defects
